# 171. Retrospective Review of Management of Acute Otitis Media in a Canadian Pediatric Emergency Department

**DOI:** 10.1093/ofid/ofae631.051

**Published:** 2025-01-29

**Authors:** Gunjan Mhapankar, Carsten Krueger, Waleed Alqurashi, Ewa Sucha, Richard Webster, Jennifer Bowes, Nicole Le Saux

**Affiliations:** Children’s Hospital of Eastern Ontario, Ottawa, ON, Canada; Alberta Children's Hospital, Calgary, Alberta, Canada; Children’s Hospital of Eastern Ontario, Ottawa, ON, Canada; Children’s Hospital of Eastern Ontario Research Institute, Ottawa, Ontario, Canada; Children’s Hospital of Eastern Ontario Research Institute, Ottawa, Ontario, Canada; Children's Hospital of Eastern Ontario, Ottawa, ON, Canada; Children's Hospital of Eastern Ontario, Ottawa, ON, Canada

## Abstract

**Background:**

Acute Otitis Media (AOM) is a leading cause of antibiotic prescription in children. Watchful waiting is the preferred strategy for AOM management for mildly ill children ≥ 2-years-old however current clinical practice varies. This study aims to compare published AOM guidelines by the Canadian Pediatric Society (CPS) with clinical practice in a Canadian academic tertiary care emergency department (ED).

**Figure:**
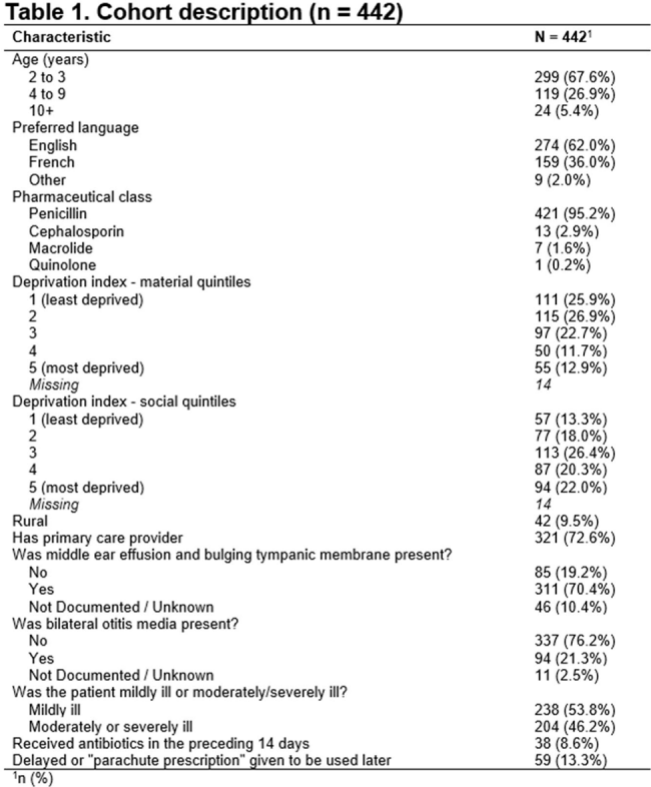

**Methods:**

We performed a retrospective review on AOM ED visits at the Children’s Hospital of Eastern Ontario (CHEO) from 1/2021 to 12/2021. Measures of non-adherence to the CPS AOM guideline were defined as follows: missed opportunity for watchful waiting, diagnostic criteria absent or not recorded, prolonged duration of treatment, lack of immediate treatment, and inappropriate agent. Additionally we explored if guideline-discordant antibiotic prescribing practices differ by geographic locale (rural vs non-rural as determined by postal code) and preferred language. The postal code was linked to a Material and Social Deprivation Index (MSDI) from INSPQ as a proxy for socio-economic status.

**Figure:**
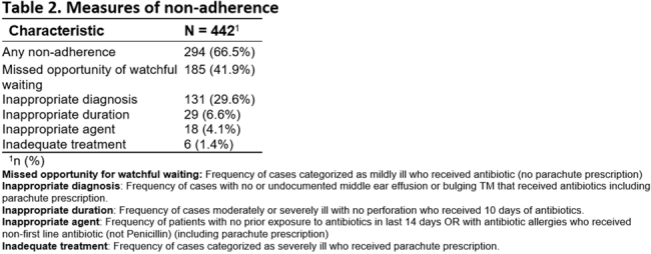

**Results:**

442 patients were included (Table 1). 66.5% patients met criteria of non-adherence (Table 2). 41.9% patients received antibiotics despite being categorized as mildly ill. 29.6% patients received antibiotics, despite having no/undocumented middle ear effusion or bulging tympanic membrane. 6.6% received longer duration of antibiotics (10 days) despite lack of complications such as perforation. 4.1% received non-penicillin antibiotics as first-line treatment. 1.4% received parachute prescriptions despite being categorized as severely ill. After controlling for language and rurality, the odds of being in any non-adherence group were not higher for patients in higher deprivation quintiles compared to those in the lowest deprivation quintile (Figure 1, 2).

**Figure 1.**
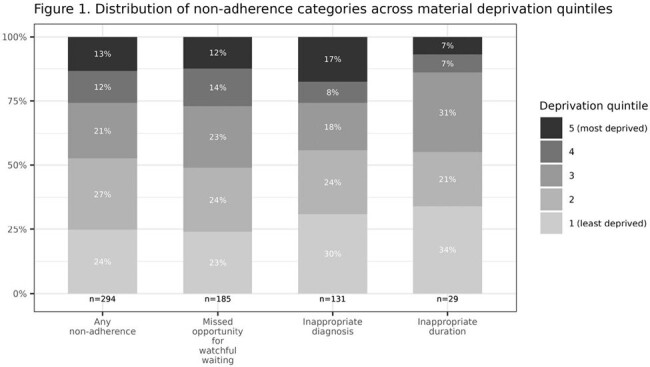
Distribution of Material Deprivation Quintiles by Non-adherence Type

**Conclusion:**

2/3^rd^ of ED patients met criteria of guideline non-adherence. Material deprivation was not a predictor of non-adherence. The two domains of watchful waiting and applying diagnostic criteria can serve as specific benchmarks to improve antibiotic prescribing practices in ED settings. Pragmatic management of AOM using current guidelines may be biased towards immediate treatment rather than timely follow-up.

**Figure 2.**
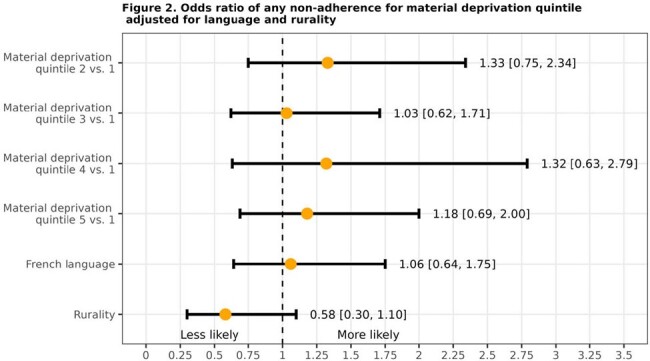
Odds ratio of any non-adherence for material deprivation quintile adjusted for language and rurality

**Disclosures:**

**All Authors**: No reported disclosures

